# Beyond physical recovery: investigating athletic identity as a mediator between social support and psychological readiness for return to sport

**DOI:** 10.1080/00049530.2024.2402424

**Published:** 2024-09-16

**Authors:** Siqi Liu, Young-Eun Noh

**Affiliations:** Faculty of Sports and Exercise Science, Universiti Malaya, Kuala Lumpur, Malaysia

**Keywords:** Psychological response, rehabilitation, sports injury, return to play

## Abstract

**Objective:**

Despite consensus on the positive relationships between social support, psychological readiness for returning to sport, and athletic identity, debate persists regarding the specific impact of these factors on athletes’ athletic identity and psychological readiness. This study investigated athletic identity as a mediator between social support and psychological readiness for returning to sport.

**Method:**

The research involved two phases. In Phase 1, four bilingual translators translated the Athletic Identity Scale and the Injury-Psychological Readiness to Return to Sport scale into Chinese using the forward-backward translation. A pilot study assessed the scales’ face validity with 30 athletes who had experienced injuries. In Phase 2, 234 injured athletes (age: *M* = 19.28, *SD* = 1.98) completed the translated measurements from Phase I and the existing Chinese version of the Multidimensional Scale of Perceived Social Support.

**Results:**

Findings showed a positive correlation between social support and both athletic identity and psychological readiness. However, athletic identity did not mediate the relationship between social support and psychological readiness.

**Conclusions:**

We recommend using various measurement tools to capture psychological readiness, particularly in the later return-to-sport stages and suggest that coaches and teammates hold welcome ceremonies to enhance psychological readiness and athletic identity through social support.

## KEY POINTS

What is already known about this topic:

(1) Social support aids in the psychological readiness for return to sport in injured athletes.

(2) After a sports injury, athletes’ levels of athletic identity tend to decline.

(3) Social support can enhance athletic identity.

What this topic adds:

(1) Athletic identity does not predict psychological readiness to return to sport after an injury.

(2) Athletic identity does not mediate the relationship between social support and psychological readiness to return to sport after a sports injury.

(3) The existing unidimensional injury-psychological readiness to return to sport scale may not capture the multidimensional constructs of psychological readiness to return to sport.

At the First World Congress in Sports Physical Therapy, 17 clinicians defined “Return to Sport” as a continuum comprising three stages: return to participation, return to sport, and return to performance (Ardern et al., 2016). “Return to participation” refers to the injured athlete’s reintegration into rehabilitation training. “Return to Sport” indicates the athlete’s engagement in training and competition at a level lower than their pre-injury performance. “Return to performance” describes the athlete’s recovery to a level of training and competition that is close to or exceeds their pre-injury performance.

This continuum encompasses a broad range of psychological and physiological recovery processes, from injured athletes participating in rehabilitation and training to returning to or surpassing their pre-injury sport level. A recent focus within this continuum is their psychological readiness aspect (Evans & Brewer, 2022). Psychological readiness for return to sport refers to an individual’s state of mental preparedness to resume sport-specific activities (Podlog et al., 2022). It can influence athletes’ perceptions of pain during the recovery process (Sanborn et al., 2022) and affect their overall recovery outcomes (Erickson et al., 2022; Peebles et al., 2021; Zhou et al., 2023).

Moreover, numerous studies have shown that psychological readiness for return to sport can serve as a predictive factor in determining whether injured athletes can successfully resume their sports (Garcia et al., 2023; Hurley et al., 2022; Lavoie-Gagne & Tanaka, 2023; Ueda et al., 2022; Webster & Feller, 2022; Webster et al., 2022). For example, psychological readiness for return to sport can predict functional outcomes at the injury site post-return (Hart et al., 2020), susceptibility to secondary injuries (Almeida et al., 2023; Faleide et al., 2021; Piussi et al., 2022), and sports performance levels (Albano et al., 2020). Therefore, some researchers suggest that the result of psychological readiness assessment should be considered as one of the factors in physicians’ decisions regarding the timing of athletes’ return to sport (Fontanez et al., 2023; Smith et al., 2021).

Given the importance of psychological readiness, exploring the contributing factors of psychological readiness appears to be highly necessary. Recent quantitative research has confirmed that perceived social support among injured athletes during the return to sport phase helps reduce their anxiety about reinjury and promotes their psychological readiness for recovery (Forsdyke et al., 2022). Additionally, qualitative interview studies with athletes who have experienced concussions, anterior cruciate ligament (ACL) ruptures, and reconstruction surgeries have identified social support as an important contributing factor to their psychological readiness for return to sport (Borman et al., 2024; Caron et al., 2021, 2023). Furthermore, social support can influence athletes’ athletic identities (Bruner et al., 2021). For example, research has shown that social support from teammates can impact athletic identity, particularly during challenging times, such as the COVID-19 pandemic (Graupensperger et al., 2020; Hagiwara et al., 2021). Athletic identity refers to how strongly an individual identifies with the role of an athlete (Brewer et al., 2022). The strength of athletic identity is associated with the occurrence of sports injuries related to overuse (Johansson et al., 2022; Martin et al., 2021), coping strategies post-injury (McGinley et al., 2024; Seguin & Culver, 2022), and levels of depression following injuries (Park et al., 2023). Moreover, the level of athletic identity can predict the practical success of injured athletes’ return-to-sport practices (Ohji et al., 2021).

However, despite the consensus among researchers on the positive relationship between social support and psychological readiness (Borman et al., 2024; Caron et al., 2021, 2023; Forsdyke et al., 2022), and the agreement on the positive relationship between social support and athletic identity (Bruner et al., 2021; Graupensperger et al., 2020; Hagiwara et al., 2021), there is inconsistency surrounding the research findings on injured athletes’ athletic identity and psychological readiness for return to sport (Loftin et al., 2023; Renton et al., 2021; Tamminen & Watson, 2022). Both qualitative and quantitative studies have consistently found that athletes often report a loss of their athletic identities after injuries (Gervis et al., 2023; Little et al., 2022; McGinley et al., 2024). Furthermore, injured athletes have revealed that the loss of athletic identity can impact their psychological readiness for return to sport (Lassman et al., 2022; Lisee et al., 2020; Mahood et al., 2020). However, Ohji et al. (2023) found no correlation between athletic identity and psychological readiness for return to sport among 105 ACL-injured patients. This contradiction between the results of qualitative and quantitative studies prompts further exploration into whether athletic identity can indeed influence psychological readiness for return to sport.

While it is established that both social support and athletic identity are significantly associated with psychological readiness for return to sport, the rationale for exploring the mediating role of athletic identity lies in understanding the underlying mechanisms of this relationship. Specifically, athletic identity may influence how social support impacts an athlete’s confidence, motivation, and mental preparedness to return to sport. By examining this mediation, we could provide a deeper understanding of how these factors interact to facilitate a successful return to sport. This could also provide a theoretical foundation for developing intervention strategies to improve psychological readiness by strengthening athletic identity. This is particularly relevant given the current lack of clear explanations regarding how certain factors facilitate psychological readiness (Podlog et al., 2022). Based on the relatively abundant qualitative research findings (Lassman et al., 2022; Lisee et al., 2020; Mahood et al., 2020), we hypothesise that athletic identity may serve as a mediator between social support and the psychological readiness of injured athletes for return to sport.

## Methods

### Study design

This study consists of two phases. In Phase 1, we invited four bilingual translators to translate the targeted measurements and convened an expert panel to check content validity. Then, a pilot study was conducted with 30 athletes who had experienced injury to assess the face validity of the translated measurements. In Phase 2, with the measurements translated and cross-culturally validated in Phase 1, we surveyed injured athletes and tested the proposed hypothesis.

### Phase 1 – Questionnaire translation and cross-cultural validation

#### Measures

##### Demographic questionnaire

This questionnaire was used to select participants for the pilot study. The questionnaire collected participants’ demographic information, such as gender, age, types of sports participation, sports level, the frequency of training cessation lasting more than seven days due to injuries, sports career, and training frequency per week.

##### Athletic Identity Measurement Scales-Third Generation

(AIMS-3 G; Brewer et al., 2022). The AIMS-3 G comprises three distinct constructs: athletic identity, athletic identity properties, and athletic identity processes. However, since the development of AIMS-3 G involved revising existing measures rather than adhering to contemporary best practices, several aspects of the process deviated from these standards. For example, the items were not pre-tested or assessed for their difficulty or representativeness of individuals’ athletic identity experiences before being used in the development sample. Consequently, the efficacy of the athletic identity properties and athletic identity processes constructs remains to be determined.

In this study, we used only the Athletic Identity Scale (AIS) from the AIMS-3 G, which measures the dimension of athletic identity. Although the AIS does not retain any items from the original 7-item Athletic Identity Measurement Scale (AIMS; Brewer & Cornelius, 2001), it demonstrates a strong correlation with the 7-item AIMS scores when examining convergent and divergent validity. This suggests that the measurement results of these two scales (i.e., AIS and AIMS) are consistent when assessing the concept of athletic identity (Brewer et al., 2022).

Moreover, in this study, we focused on the perception of athletic identity, which refers to how strongly an individual identifies with the role of an athlete (Brewer et al., 2022). Following an injury, athletes may experience a diminished perception of their athletic identity due to an extended period away from the sport (Gervis et al., 2023; Little et al., 2022; McGinley et al., 2024). Therefore, it is appropriate to use only the AIS from the AIMS-3 G to capture athletes’ perceptions of their athletic identity level during the return-to-sport phase. The AIS contains four items (e.g., “*It is in my nature to be an athlete*.”). Participants responded to these items using a 7-point Likert scale (ranging from 1 “*strongly disagree*” to 7 “*strongly agree*”), with higher scores indicating a stronger perception of their athletic identity. The AIS has demonstrated strong internal consistency, convergent validity, and discriminant validity among American college student-athletes (Brewer et al., 2022).

##### Injury-Psychological Readiness to Return to Sport

(I-PRRS; Glazer, 2009). This 6-item I-PRRS scale was designed to measure the confidence level of injured athletes for return to sport. Each item was scored on a scale (ranging from 0 “*no confidence at all*” to 10 “*complete confidence*”). The total score (ranging from 0 to 60) was calculated by summing up each item’s scores and then dividing by 10. A total score greater than or equal to 50 indicated psychological readiness for return to sport.

### The panel of experts: content validity

We assembled a panel of experts to assess the content validity of the AIS-Ch and I-PRRS-Ch scales. To qualify as an expert for this study, individuals had to meet at least one of the following criteria: 1) hold a doctoral degree in sport psychology with a record of publishing articles in internationally recognised journals on return to sport after sport injuries, 2) be a certified psychologist with at least 5 years of relevant experience, or 3) possess a coaching certification with a minimum of 5 years of practical coaching experience. Based on these criteria, we invited three individuals with extensive knowledge and experience in their respective fields to form the expert panel and provide insights and evaluations of the AIS-Ch and I-PRRS-Ch scales. Additionally, we invited the creator of the I-PRRS scale to review the backward-translated version for accuracy. Based on the creator’s suggestions, we made revisions to adapt certain items to the Chinese cultural context. For instance, to prevent subjective interpretation, we retained the phrase “100% effort” instead of using “give my best” in the third item. The demographic characteristics of the panel members are provided in Table 1.

**Table 1.** Demographic characteristics of the expert panel.

Expert	Qualified Profession	Current Position at Organization	Years of Working Experience
Expert 1	Doctor/sport psychologist	Professor	16
Expert 2	Doctor/sport psychologist	Professor	28
Expert 3	Master/Qualified coach	Head coach	15

### Participants of the pilot study

Individuals were eligible for the pilot study if they 1) were over 18 years old, 2) had a minimum of 1 year of sports training or competition experience, and 3) had sports injury experience of recovering for more than seven days. During the pilot study, 30 athletes (male = 17, female = 13, age: *M* = 19.93, *SD* = 1.11) with a history of sports-related injuries were invited to complete the AIS-Ch and I-PRRS-Ch. Table 2 presents their demographic information.

**Table 2.** Demographics of the participants for a pilot study (*N* = 30).

Demographics	Values	Percentage
Age	18 - 19	30
	20 - 21	63.3
	≥22	6.7
Training frequency (per week)	1 - 2 times	10
	3 - 4 times	60
	5 - 7 times	30
Types of sports participation	Basketball	20
	Volleyball	16.7
	Football	16.7
	Track and field	33.3
	Martial arts	13.3
Sports level	University level	30
	City level	23.3
	Province level	33.3
	National level	13.3
Sports career	1 - 2 years	23.3
	2 - 3 years	43.3
	>3 years	33.3
Training cessation frequency (≥7 days)	1 time	20
	2 times	33.3
	≥3 times	46.7

### Procedure

After obtaining ethical approval from the University of Malaya Research Ethics Committee (UM.TNC2/UMREC_2691), we invited four bilingual translators to translate the English version of the AIS and the I-PRRS into the Chinese version (AIS-Ch and I-PRRS-Ch) using the forward-backward translation method (Beaton et al., 2000). This method is particularly useful in cross-cultural research to ensure that translated instruments measure the same constructs as the original, thus maintaining validity and reliability across different languages and cultures. The process involves two main steps: 1) Forward translation. In this step, the original document (e.g., questionnaire) is translated from the source language (e.g., English) into the target language (e.g., Chinese) by a bilingual translator who is proficient in both languages. 2) Backward translation. In the next step, a second bilingual translator skilled in both languages translates the document back from the target language into the source language. This is done without reference to the original version to avoid bias.

After translating the scales, we invited an expert panel to review the back-translated version and compare them with the original English version to identify any discrepancies, differences in meaning, or cultural nuances that may have been lost or altered during the translation process. This ensures that the translated version accurately reflects the original content, meaning, and context. The panel suggested clarifying certain items. For example, “I won’t be distracted by focusing on my injury” was rephrased as “After returning to sport, my attention will not be diverted by my injury”.

To determine the measurements’ face validity, 30 adult athletes from a sports club were invited to complete the AIS-Ch and I-PRRS-Ch via Wenjuanxing (Wenjuanxing, 2023), an electronic survey platform similar to Google Forms. To prevent participants from taking the survey multiple times, we used a login system that allowed each participant to complete the survey only once. After completing the questionnaires, the first author conducted follow-up interviews with the participants through WeChat calls to inquire about their opinions on any unclear aspects of the two translated questionnaires. Based on their feedback, the first author modified the questionnaires until the participants fully understood the translated AIS and I-PRRS in the Chinese context. For example, one modification was made to the demographic section, where we asked about injury duration. Participants were unclear whether to provide the injury date or the time elapsed since the injury, particularly in a Chinese context. To address this, the first author clarified in the final version that participants should report the time elapsed since the injury, using units of days and months. Additionally, based on participants’ feedback, the first author revised the questionnaire by replacing the numerical options (1 to 7) in the AIS-Ch scale with letter options (A to G). Participants were then required to write their responses in parentheses following each question, rather than directly selecting an answer. This fill-in-the-blank style is more aligned with the common approach to answering questionnaires in China.

**Figure 1.**
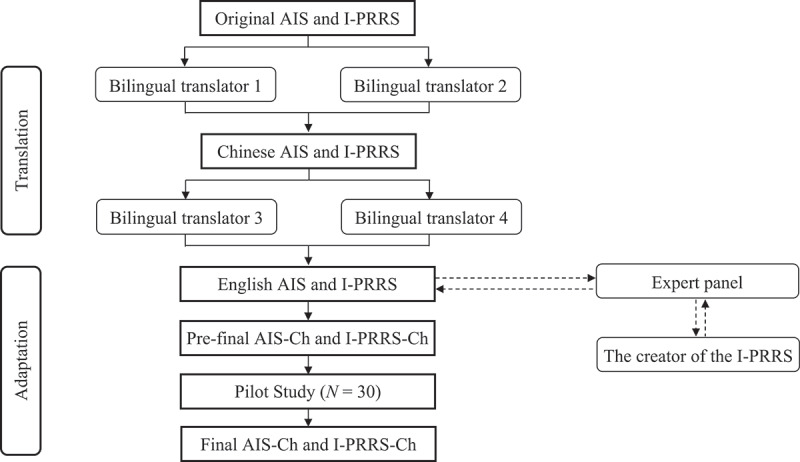
Flowchart of the translation and adaptation procedure for the AIS-Ch and I-PRRS-Ch scale.

Figure 1 shows the flowchart of the translation and adaptation processes for the AIS-Ch and I-PRRS-Ch scales.

I-PRRS = the Injury-Psychological Readiness to Return to Sport scale; I-PRRS-Ch = the Chinese Version of Injury-Psychological Readiness to Return to Sport scale; AIS = Athletic identity scale; AIS-Ch = the Chinese Version of Athletic identity scale.

### Phase 2 – Testing athletic identity as a mediator between social support and psychological readiness to return to sport

#### Participants

This study employed a purposive sampling method to recruit participants. Athletes were eligible for participation if they (1) were over 18 years old; (2) had at least one year of sports training or competition experience; (3) had been experiencing a sports injury for at least seven days (including receiving surgical and conservative treatment); (4) were in various stages of returning to sport, ranging from rehabilitation training to performance; (5) aimed to return to the same level as before the injury; and (6) were capable of reading simplified Chinese and could complete the questionnaire manually. Athletes were excluded from the study if their injuries were unrelated to sports engagement, such as those caused by rheumatic or neurological diseases.

We calculated the minimum sample size for a structural equation model as 137 with a-priori power analysis (Soper, 2023), with four unobserved variables and 22 indicator variables. The significance level, effect size, and power level were set at .05, .30, and .80, respectively, standards commonly used in social sciences research (Hair et al., 2019). A medium effect size of .30 was selected, reflecting its typical application in social sciences to balance the detection of meaningful effects with real-world utility. We distributed 272 paper questionnaires and collected 234 valid ones (male: *n* = 194, female: *n* = 40; age: *M* = 19.28, *SD* = 1.98). Table 3 indicates the participants’ demographic information.

**Table 3.** Demographics of the participants (*N* = 234).

Demographics	Values	Percentage
Age	18 - 20	79.5
	21 - 23	12.8
	24 - 26	6
	≥26	1.7
Training frequency (per week)	1 - 2 times	25.6
	3 - 4 times	44.9
	5 - 7 times	29.5
Types of sports participation	Basketball	12.0
	Volleyball	24.4
	Gymnastics	3.8
	Track and field	22.2
	Martial arts	6
	Football	31.6
Sports level	University level	28.2
	City level	55.6
	Province level	13.7
	National level	2.6
Sports career	1 - 2 years	39.3
	2 - 3 years	35.4
	>3 years	25.2
Training cessation time	1 week to 1 month	34.2
	1 month to 3 months	46.2
	3 months to 6 months	13.2
	≥6 months	6.4
Site of injury	Shoulder	1.3
	Limbs	70.1
	Waist and Abdomen	25.2
	Head	3
	Chest	0.4
Types of injury	Acute	68.4
	Overuse	31.6
Treatment method	Conservative treatment	37.1
	Surgical treatment	62.8

#### Measures

##### Demographic questionnaire

This questionnaire collected participants’ demographic information, including gender, age, type of sport, sports career length, level of competition, weekly training frequency, duration of training cessation, injury site, type of injury (i.e., acute or overuse), treatment method (i.e., conservative or surgical), whether the injury occurred during sports participation.

AIS-Ch and I-PRRS-Ch. These scales were developed in Phase 1.

##### Multidimensional Scale of Perceived Social Support

(MSPSS; Zimet et al., 1988). The MSPSS was employed to assess an individual’s perceived social support. It consists of 12 items and three dimensions: 4 items for family (e.g., “My family is willing to help me make decisions”), 4 items for friends (e.g., “I can count on my friends when things go wrong”), and 4 items for significant others (e.g., “I have a special person who is a real source of comfort”). The participants were invited to express their level of agreement with each statement on a 7-point Likert scale (ranging from 1 “*strongly disagree*” to 7 “*strongly agree*”). The total score (ranging from 12 to 84) was obtained by summing the scores from the 12 items, with higher scores indicating increased perceived social support. We used the Chinese version of the MSPSS (Wang et al., 2021), in which two dimensions (friends and significant others) were merged into a single dimension (non-family). Therefore, the Chinese version of the MSPSS contains two dimensions (family and non-family) and demonstrates a strong model fit value (goodness-of-fit index = .82) and a Cronbach’s alpha of .95 in the Chinese context, affirming its robust psychometric properties.

### Procedure

The participants were recruited offline (e.g., four sports clubs, 13 rehabilitation centres, and four hospitals) in Gansu Province in China. Offline recruitment was conducted using our informal and professional networks. We explained the purpose of the study, participation criteria, and study procedure to intermediaries, including coaches, physical rehabilitation therapists, and physiotherapists, via WeChat calls. When they expressed a willingness to assist, they were requested to distribute paper questionnaires and consent forms to potential participants who met the participation criteria and were in the return to sport process. Each participant who voluntarily engaged in this study was required to sign the provided consent form before completing the questionnaires. Subsequently, the collected questionnaires were gathered by the intermediaries and returned for data analysis. The data were collected between August 2023 and January 2024. To reduce mistakes that might occur when manually entering the collected data into a Microsoft Excel spreadsheet (Microsoft Corporation, 2018), two individuals double-checked the entries to ensure accuracy.

### Data analysis

Partial least squares structural equation modelling (PLS-SEM), used by SmartPLS version 4 (2024), was employed to test athletic identity as a mediator between social support and psychological readiness to return to sport. PLS-SEM focuses more on predicting key target constructs and understanding the predictive power of the model, which is suitable for applying this research to predict outcomes. We followed the analytical steps of the model. First, we employed confirmatory factor analysis to verify the structure of the latent constructs, ensuring that the measures were valid and reliable for subsequent use in this research. Internal consistency was evaluated using composite reliability (CR) and Cronbach’s alpha. The measurement model’s consistency and distinctiveness were validated through assessments of convergent and discriminant validity. Convergent validity was evaluated using average variance extracted (AVE) and loading values. Discriminant validity was assessed using the Heterotrait-Monotrait ratio of correlations (HTMT._85_) criterion. Second, we checked for potential collinearity issues in the structural model through the variance inflation factor (VIF). Third, we assessed the variance in the endogenous variables explained by the exogenous variables using *R*^*2*^ values. Fourth, we measured *Q*^*2*^ to assess the predictive relevance of the model. Predictive relevance indicates how well the model can predict the data points in the constructs it aims to predict. Fifth, we evaluated the effect size (*f* ^2^) to measure the impact of exogenous constructs (predictors) on endogenous constructs within the model. The effect size helps to determine the strength of the relationship between variables and whether changes in a predictor have a substantive effect on the outcome variables. Lastly, we tested the statistical significance of path coefficients to detect the correlation between exogenous variables and endogenous variables using the bootstrapping method (commonly 5,000 times). This technique assesses the precision of the path coefficients, t-values, and other statistical measures (e.g., standard errors, confidence intervals, and mediation effects).

## Results

### Testing measurement model

Table 4 presents the outcomes of the outer loadings for all indicators in the measurement model. The findings indicate that the outer loadings of all reflective measurement models exceeded 0.6. Both the CR and Cronbach’s alpha values surpassed 0.8. Additionally, all AVE values were above 0.5. These results provide substantial evidence to support the reliability and convergent validity of the measurements (Hair et al., 2019).

**Table 4.** Results for reliability and convergent validity (*N* = 234).

Construct	Item	Factor loading	Cronbach’s Alpha	CR	AVE
Family	SSA1	0.854	0.887	0.922	0.747
	SSA2	0.884			
	SSA3	0.880			
	SSA4	0.840			
Non-family	SSR1	0.796	0.900	0.920	0.591
	SSR2	0.740			
	SSR3	0.776			
	SSR4	0.673			
	SSO1	0.751			
	SSO2	0.812			
	SSO3	0.836			
	SSO4	0.755			
AIS-Ch	AI1	0.833	0.833	0.889	0.668
	AI2	0.842			
	AI3	0.852			
	AI4	0.737			
I-PRRS-Ch	IP1	0.883	0.896	0.920	0.658
	IP2	0.778			
	IP3	0.800			
	IP4	0.804			
	IP5	0.812			
	IP6	0.785			

Family and Non-family are the subscales of the Chinese version of the multidimensional scale of perceived social support; AIS-Ch = the Chinese version of the Athletic Identity Scale; I-PRRS-Ch = the Chinese version of the Injury-Psychological Readiness to Return to Sport scale.

Table 5 displays the HTMT._85_ values for each construct, with all values below the threshold of 0.85. The results provided robust confirmation of discriminant validity across all constructs (Hair et al., 2019).

**Table 5.** Correlation of latent constructs and discriminant validity (HTMT._85_ criterion).

	AIS-Ch	I-PRRS-Ch	Family	Non-family
AIS-Ch				
I-PRRS-Ch	0.116			
Family	0.255	0.085		
Non-family	0.699	0.215	0.301	

Family and Non-family are the subscales of multidimensional scale of perceived social support; AIS-Ch = the Chinese version of the Athletic Identity Scale; I-PRRS-Ch = the Chinese version of the Injury-Psychological Readiness to Return to Sport scale.

Table 6 displays the factor loadings of the second-order model of social support, indicating the relationships between the two sub-dimensions (social support from family and non-family) and overall social support. These factor loadings were utilised to calculate the second order of the Chinese version of the MSPSS.

**Table 6.** Second-order model factor loading of the Chinese version of the MSPSS.

Second-order model of social support	Factor loading	*SE*	*t* value	*p-value*
social support -> social support from family	0.580	0.006	6.821	<.001
social support -> social support from non-family	0.942	0.001	75.793	<.001

MSPSS = multidimensional scale of perceived social support.

### Testing the structural model

In this study, there were two exogenous variables: social support and athletic identity. The VIF values for these variables were 3.229 and 3.055, respectively, both falling below the threshold of 5, indicating the absence of multicollinearity issues among the predictor variable constructs (Hair et al., 2019). The adjusted *R*^*2*^ values for athletic identity and psychological readiness for return to sport were 0.358 and 0.032, respectively. Social support explained 35.8% of the variance in athletic identity. The adjusted *R*^*2*^ for psychological readiness for return to sport explained 3.2% of social support, with athletic identity as a mediator. We employed the blindfolding procedure to assess the predictive relevance of the model, revealing *Q^2^* values of 0.212 for athletic identity and 0.026 for psychological readiness for return to sport. Based on these *Q^2^* values, the predictive relevance is moderate for athletic identity, while it is relatively low for psychological readiness for return to sport (Hair et al., 2019). The *f* ^2^ results indicated a large effect size of social support on athletic identity (*f* ^2^ = 0.564), while psychological readiness for return to sport exhibited a small effect size for social support (*f* ^2^ = 0.035) (Cohen, 1992).

### Testing the mediation effects

To test the hypothesis, we employed the bootstrapping method to assess the mediation effect between social support and psychological readiness for return to sport. According to the results, the significant positive path a (*β* = 0.590, *p* < 0.001) suggested that there was a positive relationship between social support and athletic identity. In addition, the significant positive path c’ (*β* = 0.222, *p* < 0.05) indicated that there was a positive relationship between social support and psychological readiness for return to sport. However, the bootstrapping results indicated that there were no mediated effects (path b) between athletic identity and psychological readiness for return to sport (*β* = −0.049, *p* = 0.612) (see Table 7).

**Table 7.** Relative paths for the model.

Path	*β*	*SE*	*t* value	*p-value*
Path a (IV to Mediator)				
Social Support -> Athletic Identity	0.590	0.029	13.290	0.001**
Path b (Mediator to DV)				
Athletic Identity -> Return to Sport	−0.049	0.063	0.507	0.612
Path c’ (Direct effect of IV on DV)				
Social Support -> Return to Sport	0.222	0.001	2.467	0.014*

IV = Independent Variable, DV = Dependent variable, *p < .05. **p < .001.

**Figure 2.**
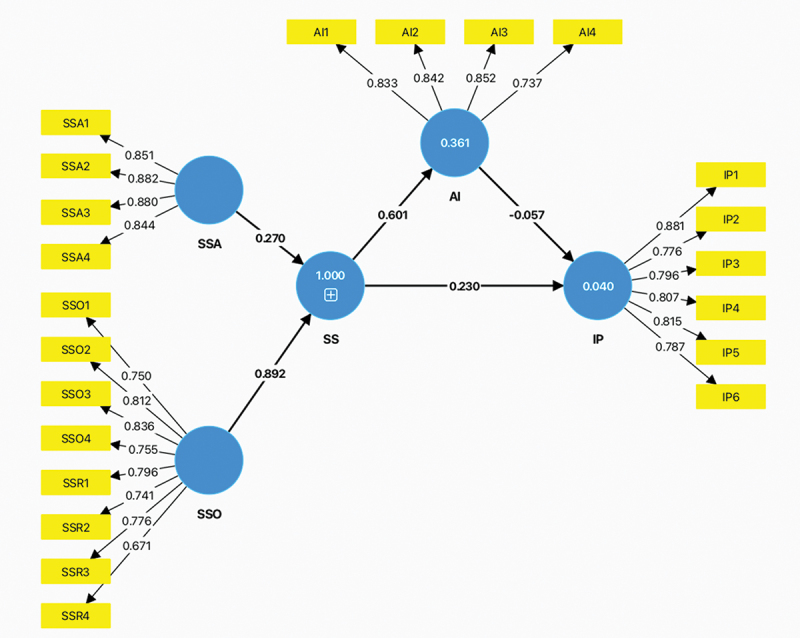
Path model.

Table 8 shows the total (direct and indirect) effects of social support on psychological readiness for return to sport. Figure 2 illustrates the proposed model, depicting social support as the independent variable and athletic identity as a mediator influencing psychological readiness for return to sport.

SS = social support; IP = psychological readiness for return to sport; AI = athletic identity; SSA = social support from family; SSO = social support from non-family.

**Table 8.** Total (direct and indirect) effects of social support on psychological readiness to return to sport.

Path	Total Effect	Direct Effect	Indirect Effect	Results
Social Support -> Return to Sport	0.192**	0.222*	−0.029	No Mediation

*p < .05; **p < .01.

## Discussion

This study aimed to investigate athletic identity as a mediator between social support and psychological readiness for return to sport following injuries. The results revealed a positive relationship between social support and athletic identity as well as between social support and psychological readiness for return to sport. However, athletic identity did not act as a mediator in the relationship between social support and psychological readiness for return to sport. The following discussion explores the possible reasons why athletic identity did not mediate the relationship between social support and psychological readiness for return to sport, while also highlighting the significant relationships between social support, athletic identity, and psychological readiness for return to sport, in comparison to previous research.

Our study found that the relationship between athletic identity and psychological readiness to return to sport was not significant, which contradicts our initial hypothesis. One possible explanation is that most participants were in the early stages of returning to sport, as indicated by our demographic data: 62.8% of the sample had undergone surgical treatment, and 80% had returned to sport within three months. At this early stage, participants might not have fully regained their pre-injury level of sport performance. Ohji et al. (2021) found that injured athletes who did not return to their pre-injury level of sport had significantly lower athletic identity compared to those who did return. This suggests that changes in athletic identity may not be prominent during the early phases of return to sport. McGinley et al. (2024) also observed a general decline in athletic identity during rehabilitation, but this decline was statistically insignificant.

Additionally, psychological readiness for return to sport tends to increase progressively from post-surgery to full return (Kostyun et al., 2021; Webster & Feller, 2022). Therefore, the lack of significant change in athletic identity during the early return-to-sport phase may account for the non-significant relationship found between athletic identity and psychological readiness in this study. This explanation aligns with the findings of Ohji et al. (2023), who reported no significant relationship between athletic identity and psychological readiness to return to sport after ACL injury. Their measurement time point was between injury and awaiting ACL reconstruction – a period during which athletic identity levels may still be low following a fresh injury. However, Ohji et al. (2021) found that only athletes who had returned to their pre-injury level of sport showed significant recovery in their athletic identity levels. Therefore, we suggest that future research include participants who are at a stage where their sport performance is close to or exceeds their pre-injury levels. This may help better capture the relationship between athletic identity and psychological readiness to return to sport.

Another reason is that there is currently no widely recognised and validated scale specifically designed to measure psychological readiness for return to sport (Evans & Brewer, 2022). To the best of our knowledge, the I-PRRS is the only scale available for measuring psychological readiness across various types of sports injuries. Recent studies have demonstrated its strong capacity to capture the concept of psychological readiness (Dunlop et al., 2023). Moreover, the I-PRRS exhibits good sensitivity across different genders and injury types (Dluzniewski et al., 2024). Therefore, it is well-suited for surveys involving a broad range of injury types in this study. However, our findings indicate that there was no relationship between athletic identity and psychological readiness for return to sport. This might be because the single – dimension I-PRRS scale focuses exclusively on assessing confidence levels during a return to sport, while overlooking other factors that may affect psychological readiness, such as emotions or mood states (Podlog et al., 2022).

Podlog et al. (2022) conducted a comprehensive narrative review of the literature from various disciplines, cultures, and languages and defined psychological readiness for return to sport across three dimensions: cognitive appraisal, affective, and behavioural components. Consequently, the single – dimensional I-PRRS may have limited the detection of significant relationships between athletic identity and psychological readiness for return to sport. Considering the potential multidimensional nature of psychological readiness, we recommend that future research simultaneously adopt multiple scales (e.g., the Fear of Return to Sport Scale, Kalatakis-dos-Santos et al., 2023) to provide a more comprehensive assessment. Alternatively, to better capture the multifaceted nature of psychological readiness, future research needs to aim at developing more comprehensive, multi-dimensional instruments that incorporate cognitive appraisal, affective, and behavioural components.

The other reason is that the AIS does not fully capture the entire concept of athletic identity. In this study, we selected only the 4-item AIS subscale of the AIMS-3 G. Similar to the AIMS used in the study by Ohji et al. (2023), the AIS focuses solely on the degree to which athletes identify with their athletic identities. Recently, Brewer et al. (2018, 2022) suggested that the measurement of athletic identity should encompass not only the concept of athletic identity itself but also the descriptive characteristics of athletic identity (athletic identity properties) and the dynamic elements that act upon and produce systematic changes in athletic identity (athletic identity processes). For example, in the context of sports injuries, injured athletes may come to realise that their sport is no longer as central as it once was, leading them to shift their priorities towards school, friends, children, or other aspects of their lives. This shift could strengthen other parts of their identity, potentially explaining why pre-injury athletic identity is an unreliable predictor of return to sport, as well as the absence of mediation. Therefore, future research could investigate the potential relationship between them using the more comprehensive construct of athletic identity with the AIMS-3 G. Such efforts could enhance our understanding of the phenomena related to athletic identity in the context of psychological rehabilitation from sports injuries.

This study found a positive relationship between social support and athletic identity during the return-to-sport phase after injury, which is consistent with previous research (Bruner et al., 2021; Graupensperger et al., 2020; Hagiwara et al., 2021). Bruner et al. (2021) found that social support promotes athletic identity in adolescent athletes. Additionally, Graupensperger et al. (2020) and Hagiwara et al. (2021) examined the impact of social support on athletic identity during the COVID-19 pandemic and highlighted that social support aided in the recovery of athletic identity for athletes returning to sport after an injury.

There was also a positive relationship between social support and psychological readiness for return to sport following injuries. The results are consistent with a previous study’s findings when they tested the mediating effect of reinjury anxiety between social support and psychological readiness for return to sport (Forsdyke et al., 2022). Based on a survey of 150 football athletes with injury experiences, Forsdyke et al. (2022) found a positive association between social support and psychological readiness for return to sport. Given these consistent findings, we recommend that future researchers develop psychological interventions aimed at enhancing the perceived level of social support among injured athletes, as such efforts could facilitate quicker psychological readiness for a return to sport. Additionally, the Chinese version of the MSPSS captures only the perceived social support of injured athletes and does not account for proactive behaviours in seeking social support (e.g., actively joining support groups related to specific issues or interests or seeking professional help). It also does not clarify how these behaviours relate to psychological readiness for a return to sport. Therefore, exploring proactive social support-seeking behaviours among injured athletes may provide a more comprehensive understanding of the relationship between social support and psychological readiness for a return to sport.

### Limitations and future directions

Although this study surveyed injured athletes while returning to the sport, there is a potential concern regarding athletes providing false reports in this research. Some athletes, eager to promptly return to sport and aware that questionnaires may influence their prospects for return to sport, may overstate their perceived psychological readiness for return to sport when completing the questionnaires distributed by coaches, physical rehabilitation therapists, and physiotherapists. This tendency could have introduced bias in this study’s survey results. Therefore, we recommend that future research utilise more objective neurocognitive measures to assess psychological readiness during the return-to-sport phase. Neurocognitive testing evaluates cognitive functions (e.g., memory, attention, reaction time, and decision-making) and their effect on physiological patterns of neural activity, which are crucial for safe and effective sport performance. Incorporating objective neurocognitive measures into the return to sport process could enhance the safety and effectiveness of the transition, ensuring that athletes are fully prepared both mentally and physically, and avoiding the ambiguous assumptions inherent in subjective measurements (Schilaty et al., 2016).

Another limitation of this study is the inclusion of athletes with varying types of injuries and different durations of time-loss, which introduces heterogeneity among the participants. For example, an injury resulting in 7 or 8 days of time-loss may provoke a much milder psychological response and have a smaller impact on athletic identity compared to an injury causing a year or more of time-loss. This variation could differently affect the athletes’ identities and their psychological readiness to return to sport. Therefore, future research should examine how different recovery processes influence various aspects of athletic identity and psychological readiness to return to sport.

Although our study did not demonstrate the mediating role of athletic identity in the relationship between social support and psychological readiness for return to sport, other psychosocial factors (e.g., self-efficacy, motivation, coping skills, resilience, or group cohesion) might mediate this relationship. Alternatively, factors such as gender, age, type of injury, or duration of rehabilitation could influence the relationship between social support, athletic identity, and psychological readiness. Future research should explore the impact of these variables, potentially through multi-group analysis or moderation models, to gain a more nuanced understanding of their roles in the return-to-sport process.

### Applied implications

The results of the current study can offer practical suggestions for application. For injured athletes, practitioners could implement social support from family, teammates, friends, and coaches through care and visits during the recovery phase. Additionally, during the return to sport phase, coaches and teammates could hold welcome ceremonies for returning athletes. This type of social support could help restore and enhance the athletic identity of injured athletes and improve their psychological readiness for return to sport. Such efforts could promote better athletic performance post-return (Van Haren et al., 2023) and reduce the likelihood of re-injury after return to sport (Faltstrom et al., 2021). However, it is important to note that these efforts must consider each individual’s preferences to avoid placing unnecessary pressure on the returning athlete.

## Conclusion

This study examined the psychosocial factors that may influence psychological readiness for return to sport. We found that athletic identity did not mediate the relationship between social support and psychological readiness for return to sport. Future studies could explore this relationship by using various measurements to capture the possible multidimensional concept of psychological readiness for return to sport, along with using AIMS-3 G instead of AIS. These efforts may help uncover the mediating role of athletic identity and the relationship between social support and psychological readiness for return to sport.

## Data Availability

The data that support the findings of this study are available from the corresponding author, [N, Y], upon reasonable request.
